# Current status and influencing factors of job crafting ability in operating room senior nurses: a cross-sectional study

**DOI:** 10.3389/fpsyg.2025.1692813

**Published:** 2025-11-28

**Authors:** Qiwen Lian, Weizhao Huang, Tingting Guo, Binfei Li, Xiaoping Luo

**Affiliations:** 1 Department of Surgical Anesthesiology-Division 1, Zhongshan People's Hospital, Zhongshan, Guangdong, China; 2 Cardiothoracic Surgery, Zhongshan People's Hospital, Zhongshan, Guangdong, China

**Keywords:** operating room senior nurses, job crafting, professional identity, organizational support, nursing

## Abstract

**Background:**

Senior nurses are pivotal to healthcare quality but face heightened burnout and career stagnation, especially in high-stakes environments like the operating room (OR). Job crafting, a proactive behavior where employees reshape their work, is a potential strategy to enhance their resilience and engagement. However, evidence on job crafting and its drivers among OR senior nurses remains limited.

**Aims:**

To investigate the job crafting ability of OR senior nurses and its influencing factors within a specific regional context, providing a basis for targeted interventions.

**Design:**

A cross-sectional study.

**Methods:**

In February–May 2025, 210 OR senior nurses from four hospitals in Zhongshan, China (including 2 tertiary hospitals, 1 secondary hospital, and 1 primary care institution) were selected via convenience sampling. Data were collected using general information, job crafting, organizational support, and professional identity scales. Analyses included descriptive statistics, *t*-test, ANOVA, Spearman correlation, and multiple linear regression.

**Results:**

The job crafting score (21 items) averaged 68.04 ± 22.876, indicating a lower-middle level. Multiple linear regression revealed that organizational support (β = 0.679, *P* < 0.001), professional identity (β = 0.273, *P* < 0.001), hospital grade, and employment type were significant predictors, collectively explaining 75.7% of the variance in job crafting scores.

**Conclusion:**

Among OR senior nurses in the surveyed hospitals of Zhongshan, job crafting was at a lower-middle level and significantly influenced by organizational support, professional identity, hospital grade, and employment type. The findings, primarily reflective of a female-dominated sample within this specific context, suggest that nurse managers should develop strategies targeting these factors to enhance job crafting. However, the generalizability of these conclusions to other regions or different gender compositions requires further investigation.

## 1 Introduction

Job crafting, as proposed by Wrzesniewski and Dutton (2001), refers to the proactive behaviors through which employees adjust their work tasks, interpersonal relationships, and cognitive perceptions to better align their jobs with their own values, interests, and abilities. Cullinane et al. (2017) further classified job crafting into three dimensions: task crafting (modifying the type or scope of tasks), relational crafting (choosing with whom one interacts at work), and cognitive crafting (reframing the meaning of work), highlighting its nature as a continuous process. Notably, job crafting has been recognized as a key antecedent of job engagement, directly shaping how employees experience and invest themselves in their work (Bakker et al., 2016; Iida et al., 2024). In the context of nursing, job crafting is not only associated with individual nurses' wellbeing but also influences overall care quality and team stability. In China, with the ongoing advancement of high-quality nursing service reforms, senior nurses are facing increasing pressures such as role overload and career stagnation, making their capacity for job crafting essential for sustaining the nursing workforce. However, existing research has long overlooked the unique challenges and distinct experiences of this group, which underscores the significance of this study.

Senior nurses are clinical nurses who have been engaged in clinical nursing for more than 10 years and have rich clinical experience, strong working ability, as well as certain unit management ability (Jang et al., 2019). Senior nurses assume a key role in the nursing team due to their rich clinical experience and stable professional competence. However, being in a high-pressure environment for a long period of time also causes them to face problems such as increased burnout and decreased job satisfaction, thereby affecting their career development and willingness to leave (Helaß et al., 2025; Rahnfeld et al., 2023).

For operating room senior nurses, these challenges are further amplified by the unique demands of surgical settings: (1) Rigid workflow constraints (e.g., fixed surgical procedures and sterile protocols) limit opportunities for task restructuring; (2) High-stakes environments reduce autonomy in cognitive reshaping due to standardized safety requirements; (3) Interdisciplinary team dynamics complicate relational crafting, as roles are hierarchically defined during surgeries (Dai et al., 2025). Consequently, job crafting for this group requires tailored strategies addressing these contextual barriers.

Studies show that the incidence of job burnout among senior nurses is higher compared with that among junior nurses (Zhou and Gong, 2015). This is closely related to its heavy burden, few opportunities for skills training, and insufficient support for career development (Luan et al., 2017; Valencia and Raingruber, 2010; Clendon and Walker, 2016). Due to the lack of systematic career planning guidance and imperfect promotion mechanisms, it is difficult for senior nurses to make further breakthroughs in their career development, and their sense of professional value and achievement gradually weakens. Kotzabassaki and Parissopoulos (2003) pointed out that this limited career development space not only weakens the motivation of senior nurses to reshape their jobs, but also may become an important inducement to aggravate job burnout.

In this context, job crafting, as a bottom-up self-regulation strategy, provides senior nurses with the opportunity to redefine the content and significance of their work (Chu et al., 2022). By proactively optimizing the structure of work tasks, improving interpersonal interactions, and reconfiguring cognitive perspectives on work, senior nurses are able to find a more personalized and meaningful career path in a demanding work environment (Han, 2023). Thereby enhancing occupational resilience and psychological adaptability (Hornung, 2019; Niessen et al., 2016; Roczniewska and Bakker, 2021).

Research shows that there is a significant correlation among job crafting, perceived organizational support, and personal professional identity. It was found that the stronger the perceived organizational support, the higher the ability to actively adjust the work content and optimize the work style, and there was a significant positive correlation between the two (Romeo et al., 2023; Khattab et al., 2023). This suggests that organizational support plays an important role in facilitating job crafting. In addition, the findings of Xie et al. (2024) indicate that professional identity has a direct influence on job crafting, i.e., the stronger the nurses' professional identity, the higher their ability to remodel their jobs. A strong sense of professional identity may enhance nurses' recognition of their own professional value, which is more conducive to their ability to manifest their own job crafting in the complex and changing clinical environment.

Current research primarily examines job crafting among new nurses (Duan et al., 2024) and nurses in different departments (Yang et al., 2024; Harbridge et al., 2023), as well as related mediating effects (Topa and Aranda-Carmena, 2022; Park and Ha, 2025), Crucially, no studies have distinguished between the challenges of senior and junior nurses or analyzed the unique barriers to job crafting faced by operating room senior nurses. This gap means current interventions cannot address the career bottlenecks and contextual constraints specific to operating room senior nurses, failing to support their long-term career development or stabilize surgical nursing teams.

To fill this gap, this study focuses exclusively on operating room senior nurses. Its objectives are to: (1) Investigate the current status of their job crafting ability; (2) Identify key influencing factors, including sociodemographic characteristics, professional identity, and organizational support, that are specific to their role and work environment; (3) Provide targeted strategies to enhance their professional identity and organizational support. By addressing these objectives, this study aims to offer a theoretical basis for optimizing interventions, promoting the sustainable career development of operating room senior nurses, and strengthening the stability of surgical nursing teams.

## 2 Methods

### 2.1 Aims

The study aimed to investigate the current status of job crafting ability among operating room senior nurses and to determine surgery-specific factors affecting their job crafting ability through socio-demographic characteristics, professional identity, and perceived organizational support in high-acuity surgical environments.

### 2.2 Design

This was a cross-sectional study that included a descriptive survey. Our study was reported based on the Report of Observational Studies in Epidemiology (STROBE) Cross-sectional Research Inventory (Dewidar et al., 2025).

### 2.3 Participants

A total of 210 senior nurses from four hospitals in Zhongshan City, Guangdong Province, China, were selected in February 2025 using convenience sampling method as the study subjects. Inclusion criteria: ①Operating room nurses with active licensure; ②10 years of working hours; ③Informed consent and voluntary participation in this study. The exclusion criteria were: (1) nurses in training, rotation, and training program.

### 2.4 Sampling

According to Kendall's sample size estimation method (Bacchetti and Leung, 2002), the sample size is at least 5–10 times the number of independent variables. Furthermore, to account for potential non-responses, the sample size calculation should be increased by an additional 20%. The formula is *N* = [(5–10) × *n*]/(1–0.2). This study encompassed 22 variables; thus, using the formula *N* = (22 × 5)/0.8, the minimum required sample size was calculated to be 138. In total 210 cases were finally included in this study.

### 2.5 Measures

#### 2.5.1 General information questionnaire

The general information questionnaire was designed by the research team in accordance with the study objectives and in conjunction with elements related to work remodeling in previous literature studies, including age, gender, education, hospital grade, marital status, professional title, position, Employment Status, years of experience, monthly income, number of night shifts, Working hours per week, and self-perceived health status.

#### 2.5.2 Job crafting scale

The job crafting scale (JCS) was developed by Tims et al. (2012) and revised by Chinese scholar Liao (2013). It contains 4 dimensions, namely, increase structural work resources (5 entries), increase social work resources (5 entries), increase challenging work demands (5 entries), and reduce hindering work demands (6 entries), with a total of 21 entries. Items were rated on a 5-point Likert scale (1 = strongly disagree to 5 = strongly agree), and the higher the score, the better the job crafting behavior, and the Cronbach's alpha coefficient of the scale was 0.820.The Cronbach's coefficients for the four dimensions were 0.838, 0.733, 0.722, and 0.551.

#### 2.5.3 Nurses' sense of organizational support scale

Developed by Chinese scholars Wang et al. (2014), this is a one-dimensional scale with a total of 15 items. It is a one-dimensional scale with 15 items. It is scored on a 5-point Likert scale, with 1–5 representing “strongly disagree” to “strongly agree,” and the higher the score, the higher the degree of nurses' sense of organizational support, and the Cronbach's alpha coefficient of this scale is 0.957.

#### 2.5.4 Nurses' occupational identity rating scale

Developed by Chinese scholars Liu et al. (2011), the scale consisted of 5 dimensions of career cognitive appraisal, career social skills, career social support, career frustration, and career self-reflection, with a total of 30 entries. Each entry was scored on a 5-point Likert scale with a total score of 30–150 points. 30–60 points were categorized as low group, 61–90 points were categorized as low group, 91–120 points were categorized as medium group, and 121–150 points were categorized as high group, with higher scores indicating higher nurses' occupational identity. The Cronbach's alpha coefficient of the scale was 0.938. The Cronbach's coefficients for the five dimensions were 0.911, 0.824, 0.763, 0.830, and 0.72.

### 2.6 Data collection

The electronic link of the questionnaire was created through the questionnaire star platform, and the consent of the nursing departments of the four hospitals was obtained for the distribution of the questionnaire, and a unified guideline was adopted to explain the purpose, content and filling method of the survey. In the process of filling out the questionnaire, if the filler omits to fill in the situation, after he/she clicks submit, there will be a prompt to fill in the questionnaire, and the questionnaire can only be successfully submitted after he/she fills in the complete form, which ensures the completeness of the questionnaire; and the background settings can only be filled in once by the same account, so as to avoid the filler from filling in the questionnaire repeatedly. Based on the principle of voluntariness, the survey respondents fill out the questionnaire independently. A total of 236 questionnaires were recovered, excluding 26 questionnaires with obvious regularity of answers (such as all questionnaire options are consistent) and irregularities in filling out the questionnaires, 210 valid questionnaires were recovered, with an effective recovery rate of 88.9%.

### 2.7 Ethical considerations

This study was approved by the Ethics Committee of Zhongshan People's Hospital (Ethics Approval No. SFYLL-KY-PJ-2025-040). The study was conducted in accordance with the Declaration of Helsinki and its subsequent amendments or similar ethical standards. All participants signed an informed consent form and were given an explanation of the study's importance and goal by the researcher before the official investigation. Participants were told there would be no consequences if they chose to leave the research at any point. The researcher pledged to maintain the complete confidentiality and anonymity of the participants' personal information.

### 2.8 Data analysis

Data entry and analysis were done using SPSS 27.0 software, and the recovered surveys were numbered. Normally distributed measurements were statistically described using (*x* ± S); one-way ANOVA was performed using the *t*-test and ANOVA; correlations between variables were analyzed using spearman's rank correlation analysis; and multifactorial analyses were performed using multiple linear regression analysis. The test level α = 0.05, *P* < 0.05 indicates that the difference is statistically significant.

## 3 Results

### 3.1 General characteristics

A total of 210 operating room senior nurses from four hospitals in Zhongshan were surveyed. Of which 173 cases (82.4%) were female and 37 (17.6%) were male; 86.2% of them were aged between 40 and 50 years old, and 70 nurses (33.3%) were nurses with associate degree; 85 nurses (40.5%) were nurses with bachelor's degree; and 55 nurses (26.2%) were nurses with master's degree of postgraduate studies. The grade of the hospitals they worked in was 71 nurses (33.8%) in Secondary hospitals; 83 nurses (39.5%) in Tertiary Grade Hospitals and 56 nurses (26.7%) in Primary care institutions (Note: Primary care institutions refer to grassroots medical institutions, including small surgical institutions such as dental hospitals.). Positions assumed were primary nurse in 70 cases (33.3%); nursing team leader in 57 cases (27.1%); deputy head nurse in 42 cases (20%); head nurse in 30 cases (14.3%); unit nurse manager in 8 cases (3.8%); and director of nursing in 3 cases (1.4%). The employment type was 120 cases (57.1%) for permanent nurses; 90 cases (42.9%) for contract-based nurses. When comparing the job crafting scores of senior nurses with different gender, age, hospital grade, position, employment type, and self-perceived health status, the results showed that the differences were all statistically significant (*P* < 0.05), as shown in Table 1.

**Table 1:** Participants' general characteristics (*N* = 210).

**Variables**	***n* (%)**	**Job crafting (mean ±SD)**	***t*/*F***	***p***
**Gender**				
Male	37 (17.6)	56.57 ± 20.29	3.447	**< 0.001** ^**^
Female	173 (82.4)	70.49 ± 22.70		
**Age (years)**				
40–45	101 (48.1)	63.77 ± 22.19	**3.558**	**0.030** ^*^
46–50	80 (38.1)	72.55 ± 22.68		
>50	29 (13.8)	70.45 ± 23.79		
**Education**				
Associate degree	70 (33.3)	65.75 ± 22.09	0.392	0.676
Bachelor's degree	85 (40.5)	68.52 ± 22.07		
Master's degree	55 (26.1)	69.26 ± 23.87		
**Hospital grade**				
Tertiary hospitals	56 (26.7)	75.54 ± 20.53	**13.817**	**< 0.001** ^**^
Secondary hospitals	83 (39.5)	69.98 ± 22.73		
Primary medical institutions	71 (33.8)	55.66 ± 21.16		
**Marital status**				
Married	175 (83.3)	68.46 ± 22.52	0.358	0.699
Unmarried	19 (9.0)	63.79 ± 24.98		
Divorced, widowed	16 (7.6)	68.50 ± 25.24		
**Professional title**				
Middle level	94 (44.8)	68.84 ± 23.16	0.108	0.897
Deputy senior title	91 (43.3)	67.49 ± 22.64		
Senior title	25 (11.9)	67.00 ± 23.49		
**Position**				
Primary nurse	70 (33.3)	60.79 ± 23.15	**6.582**	**< 0.001** ^**^
Nursing team leader	57 (27.1)	69.98 ± 20.84		
Deputy head nurse	42 (20.0)	69.21 ± 25.86		
Head nurse	30 (14.3)	74.37 ± 20.71		
Unit nurse manager	8 (3.8)	77.33 ± 11.72		
Director of nursing	3 (1.4)	84.25 ± 7.94		
**Employment status**				
Permanent nurses	90 (42.9)	73.71 ± 20.87	**3.233**	**< 0.001** ^**^
Contract-based nurses	120 (57.1)	63.78 ± 23.47		
**Years of experience**				
10–15	33 (15.7)	64.12 ± 24.37	0.676	0.609
16–20	93 (44.3)	67.11 ± 22.18		
21–25	54 (25.7)	73.50 ± 22.29		
26–30	18 (8.6)	70.37 ± 23.27		
>30	12 (5.7)	67.33 ± 24.38		
**Monthly income (yuan)**				
≤ 5,000	21 (10.0)	64.81 ± 23.82	3.730	0.053
5,000–10,000	122 (58.1)	64.45 ± 23.21		
10,000–15,000	64 (30.5)	75.83 ± 19.97		
>15,000	3 (1.4)	70.33 ± 32.13		
**Number of night shifts (months)**				
0–3	74 (35.2)	74.20 ± 22.66	2.602	0.077
4–7	96 (45.7)	68.50 ± 23.19		
>7	40 (19.0)	64.11 ± 22.08		
**Working hours per week (hours)**				
< 40	55 (26.2)	63.36 ± 18.89	2.167	0.173
40–50	101 (48.1)	70.54 ± 23.53		
>50	54 (25.7)	68.11 ± 24.91		
**Self-perceived health status**				
Good	27 (12.9)	81.30 ± 22.99	**8.038**	**< 0.001** ^**^
Better	50 (23.8)	77.24 ± 19.45		
Fair	90 (42.9)	62.86 ± 22.06		
Poor	38 (18.1)	61.47 ± 21.59		
Very Poor	5 (2.4)	47.60 ± 18.77		

Bold values indicate *p* < 0.05; symbol ^*^indicates *p* < 0.05, and ^**^indicates *p* < 0.01, all representing statistically significant differences.

### 3.2 Job crafting scores of operating room senior nurses

This section describes the scores of operating room senior nurses on each dimension of job crafting. The total job crafting score consists of 21 items, with an average score of 68.04 ± 22.876. The average scores of the four dimensions are as follows: 4.03 ± 0.976 points for the increasing structural work resources, 3.75 ± 0.857 points for increasing social work resources, 3.31 ± 0.922 points for increasing challenging work demands, and 4.22 ± 0.794 points for reducing obstructive work demands, as shown in Table 2.

**Table 2:** Job crafting scores of operating room senior nurses (*N* = 210).

**Total score and dimensions**	**Score range foreach entry**	**Number of entries**	**Mean total scores (mean ±SD)**
Total score of job crafting	21–105	21	68.04 ± 22.87
Increase structural work resources	1–5	5	16.92 ± 5.96
Increase social work resources	1–5	5	15.90 ± 5.70
Increase challenging work demands	1–5	5	16.19 ± 5.61
Reduce obstructive work demands	1–5	6	19.02 ± 6.56

### 3.3 Correlation analysis of organizational support level, professional identity, and job crafting ability of operating room senior nurses

Spearman's rank correlation analysis showed that the level of job crafting was significantly and positively correlated with the level of professional identity (*r* = 0.843, *p* < 0.001) and organizational support (*r* = 0.742, *p* < 0.001). Additionally, professional identity was also significantly and positively correlated with organizational support (*r* = 0.754, *p* < 0.001), as shown in Table 3.

**Table 3:** Correlation of organizational support level, professional identity with job crafting ability in operating room senior nurses (*N* = 210).

**Item**	**Job crafting**	**Professional identity**	**Organizational support**
Job crafting	1.000		
Professional identity	0.843^**^	1.000	
Organizational support	0.742^**^	0.754^**^	1.000

^**^p < 0.01.

### 3.4 Multiple linear regression analysis of factors related to job crafting ability in operating room senior nurses

A multiple linear regression analysis was conducted on the total job crafting scores of operating room senior nurses (inclusion criterion α = 0.05, exclusion criterion α = 0.10), with variables showing statistical significance in univariate analysis and those related to job crafting set as independent variables. The independent variables were assigned as shown in Table 4.

**Table 4:** Independent variable assignment methods (*N* = 210).

**Independent variable**	**Assignment method**
Gender	Female = 1, Male = 2
Age (years)	40–45 = 1, 46–50 = 2, >50 = 3
Hospital grade	Tertiary hospital = 1, Secondary hospitals = 2, Primary medical institutions = 3
Position	Primary nurse = 1, Nursing team leader = 2, Deputy head nurse = 3, Head nurse = 4, Unit nurse manager = 5, Director of nursing = 6
Employment status	Permanent nurses = 1, contract-based nurses = 2
self-perceived health status	Good = 1, Better = 2, Fair = 3, Poor = 4, Very Poor = 5
Organizational support	Substitute the original value
Professional identity	Substitute the original value

The results of the multiple linear regression analysis indicated that organizational support, professional identity, hospital grade, and employment type were the main factors influencing the job crafting ability of operating room senior nurses (*p* < 0.05). Specifically, nurses in tertiary hospitals permanent nurses, those with high organizational support, and those with high professional identity had positive effects on job crafting among operating room senior nurses, while nurses in basic medical institutions and contract-based nurses significantly inhibited job crafting ability. Other variables (such as gender, age, and position) showed no statistical significance (^*^p^*^ > 0.05), as shown in Table 5. To further clarify the applicability of findings to female nurses (the majority group in our sample), we conducted a stratified multiple linear regression analysis for female participants (*n* = 173). In Model 1 (controlling for hospital grade and job nature), these demographic variables explained 17.2% of the variance in job crafting (*F* = 17.683, *p* < 0.001). After incorporating core variables (organizational support, professional identity) in Model 2, the explained variance increased substantially to 83.9% (Δ*R*^2^ = 0.667, *p* < 0.001), with both core variables showing significant positive effects on job crafting (β = 0.382 for organizational support, β = 0.509 for professional identity, both *p* < 0.001). Although moderate multicollinearity existed between core variables [variance inflation factor (VIF) ≈ 6.4–6.8], it did not severely interfere with model stability. These results confirmed that the associations between core factors and job crafting are robust in female nurses.

**Table 5:** Multiple linear regression analysis of job crafting in operating room senior nurses (*N* = 210).

**Variable**	***B***	**SE**	**β**	***t***	***p***	**95% CI for B**		**VIF**
						**Lower limit**	**Upper limit**	
Constant	19.096	7.356		2.596	0.010^*^	4.591	33.602	
Gender	−3.066	2.152	−0.051	−1.425	0.156	−7.31	1.178	1.110
Age	0.528	1.151	0.016	0.459	0.647	−1.742	2.798	1.097
Hospital grade	−2.273	1.092	−0.077	−2.082	0.039^*^	−4.425	−0.12	1.179
Position	−0.025	0.657	−0.001	−0.037	0.97	−1.321	1.272	1.121
Employment status	−4.237	1.628	−0.092	−2.602	0.010^*^	−7.448	−1.026	1.072
Self–perceived health status	0.719	0.894	0.031	0.804	0.422	−1.044	2.483	1.263
Organizational support	0.418	0.079	0.278	5.274	< 0.001^**^	0.262	0.574	2.380
Professional identity	0.467	0.045	0.599	10.313	< 0.001^**^	0.377	0.556	2.897

*R*^2^ = 76.5%, adjusted *R*^2^ = 75.7%; *F* = 94.103, *p* < 0.001.

^*^p < 0.05. ^**^p < 0.01.

## 4 Discussion

### 4.1 The need to further enhance job crafting abilities among operating room senior nurses

The results of this study indicate that the sampled operating room senior nurses from four hospitals in Zhongshan scored (68.04 ± 22.87) in job crafting, which is significantly lower than the scores reported by Dai et al. (2024) in their survey of junior clinical nurses. It should be noted that the generalizability of this finding is limited to the specific context of the participating hospitals. This lower score was observed in conjunction with environmental constraints specific to surgical settings, career stagnation, and psychological barriers prevalent in these institutions. The highly protocol-driven operating room environment confines experienced nurses to standardized roles (e.g., instrument management), which is associated with limited task autonomy and opportunities for innovation. Long-term involvement in repetitive tasks is linked to the solidification of professional roles. Compared to junior nurses who receive diverse skill training opportunities in clinical settings, senior nurses in these settings face limited career advancement (Uthaman et al., 2016), which correlates with suppressed innovative capabilities and willingness to change, thereby corresponding to lower internal motivation for job crafting. Furthermore, operating room senior nurses commonly experience job burnout (Luan et al., 2017).

As core members of clinical nursing, they often assume management and teaching responsibilities within their departments, such as quality control, education, and training (Blakeley and Ribeiro, 2008). Additional duties in clinical teaching and research further increase their workload to some extent (Xu and Fan, 2023). Coupled with limited promotion channels in the nursing profession, some senior operating room nurses tend to maintain the status quo in their career development (Zhong et al., 2024). In summary, the lower job crafting ability among these nurses in the studied context co-occurs with multiple factors, including career development bottlenecks, physical and mental exhaustion, and a lack of promotion opportunities. Future efforts should focus on optimizing career development pathways, establishing flexible work systems, and stimulating job crafting potential among operating room senior nurses, though the applicability of these recommendations beyond similar hospital settings requires further verification.

### 4.2 Job crafting among operating room senior nurses is influenced by multiple factors

#### 4.2.1 Hospital grade and employment type are important factors influencing job crafting among operating room senior nurses

The results of this study reveal that operating room senior nurses in tertiary hospitals within our sample generally exhibit higher job crafting ability than those in primary medical institutions. This difference is concomitant with disparities in resource allocation, career development platforms, and organizational support across hospital levels (Liu et al., 2023). In China, hospitals are classified into three tiers (World Health Organization, 2015), with higher-level hospitals possessing more advanced equipment, better staffing, and higher-quality medical care than lower-level institutions (Gong, 2014).

As high-level medical facilities, tertiary hospitals provide operating room senior nurses with more comprehensive career development systems and specialized training pathways. Exposure to complex procedures is related to continuous skill upgrading and role adaptation. These hospitals typically have well-established nursing management systems, specialized development trajectories, and continuing education mechanisms, which are associated with more opportunities for senior nurses to engage in diverse roles such as teaching, research, and quality management. This encourages them to proactively adjust task content and reframe role perceptions, facilitating an alignment between personal and organizational goals (Huang et al., 2023; Li et al., 2019). Moreover, senior nurses in higher-level institutions generally benefit from greater job stability, stronger organizational support, and more robust training systems (Cheng et al., 2020), all of which are positively correlated with self-efficacy and autonomy, thereby higher job crafting behaviors.

In contrast, primary care institutions often limit surgical nurses to repetitive minor procedures with standardized workflows. Narrow promotion channels and low case complexity are linked to reduced opportunities for skill renewal, accelerated burnout, and diminished motivation for job crafting (Wang et al., 2020; Park et al., 2019). Additionally, this study found that permanent nurses exhibited significantly higher levels of job crafting than contract-based nurses, consistent with the findings of Yuan et al. (2024). Due to poorer job stability, limited promotion opportunities, and unequal benefits, contract nurses are more susceptible to job burnout and a weaker sense of professional value (Sun et al., 2023; Zhang et al., 2015). As a result, they are more likely to adapt passively rather than proactively when facing work pressure, which corresponds to lower job crafting ability. It is worth noting that the observed associations in the aforementioned research findings should be interpreted in the context of the specific background of the sampled hospitals in the Zhongshan region. Therefore, managers should pay greater attention to supporting contract nurses and provide career development opportunities to enhance their job crafting levels.

#### 4.2.2 Operating room senior nurses with a stronger sense of organizational support exhibit higher levels of job crafting

This study demonstrates a significant positive correlation between perceived organizational support and job crafting levels among senior nurses in our sample, consistent with the results of Khattab et al. (2023). Additionally, Ahmad et al. (2022) indicated that when nurses perceive organizational attention and support, they can improve both the quality of care and job satisfaction—the latter of which is significantly positively correlated with job crafting (Rudolph et al., 2017).

The Job Demands-Resources (JD-R) model, originally proposed by Demerouti et al. (2001), posits that all jobs comprise two key components: job demands and job resources, which collectively influence employee wellbeing and performance. Within this framework, perceived organizational support is identified as a critical job resource. Excessive job demands can lead to burnout when they exceed an individual's adaptive capacity. In contrast, job resources, such as participation in decision-making and organizational support, are associated with enhanced work engagement, mitigated disengagement, and improved overall performance and wellbeing. Specifically, organizational support serves as a pivotal resource that not only buffers the adverse effects of job demands but also strengthens job stability and security. This, in turn, is linked to increased motivation for proactive behaviors like job crafting, and corresponds to the psychological capital needed for senior operating room nurses to adapt their roles and optimize work tasks.

When senior operating room nurses feel valued and supported by their organization, their willingness and ability to actively adjust work roles and improve processes are significantly enhanced. However, some healthcare institutions currently lack well-constructed organizational support systems, which adversely affects the professional vitality and innovative potential of senior nurses.

A qualitative interview study involving nurses with over 30 years of experience found that some hospitals have not established comprehensive career development support systems, leading to insufficient motivation for professional growth among senior nurses (Sun et al., 2024). The lack of perceived organizational support not only weakens job crafting motivation but also exacerbates job burnout, becoming a key constraint on career continuity (Zhu and Li, 2022). A survey of 50 senior nurses in Saudi Arabia highlighted that they expressed a need for better organizational support, including inclusive career development opportunities, fair promotion policies, and training programs addressing ageism (Mohamed and Shaban, 2024).

Therefore, it is recommended to enhance job crafting among senior nurses through structured organizational support strategies. McDonald et al. (2010) proposed a collaborative mentoring system that pairs senior and junior nurses in one-on-one mentoring groups. This approach leverages the experience of senior nurses while providing them with role expansion opportunities, strengthening professional identity, alleviating burnout, and enhancing self-efficacy in job crafting. Additionally, establishing multidisciplinary teams centered on specialized nursing can create professional development pathways for senior nurses (Zhang et al., 2017). This not only enhances professional value through role empowerment but also optimizes resource allocation via team collaboration, thereby boosting intrinsic motivation.

Nursing managers should implement flexible scheduling systems aligned with fluctuations in surgical volume, such as adjustable shifts or part-time arrangements. This can help reduce the physical and mental strain caused by long-term high-intensity work, mitigate work-family conflict, and create opportunities for job crafting. Furthermore, senior nurses should be encouraged to take leadership roles in optimizing surgical coordination workflows and developing training modules for new staff, thereby expanding their professional impact.

Moreover, nursing managers should involve senior nurses in decision-making processes, including management meetings, workflow redesign discussions, and emergency protocol development. Their frontline expertise is crucial for identifying efficiency barriers and improving surgical safety and flow. Targeted training should also be provided to enhance their leadership in surgical innovation, helping them align their specialized experience with emerging trends such as robotic surgery, minimally invasive techniques, and high-value consumables management. This will strengthen their adaptability in a rapidly evolving environment and open new pathways for role innovation.

#### 4.2.3 Operating room senior nurses with high levels of professional identity exhibit relatively higher levels of job crafting

This study shows a significant positive correlation between professional identity and job crafting levels among operating room senior nurses in the investigated cohort. Existing research suggests that professional identity is associated with job crafting behaviors, which in turn correlates with work engagement and ultimately is linked to workplace wellbeing (Zhai et al., 2023). This finding aligns with the studies by Pang et al. (2023) and Doran et al. (2015).

The stronger the professional identity among operating room senior nurses, the greater their capacity to adapt surgical workflows, optimize instrument management techniques, and reframe intraoperative roles. This indicates that professional identity serves not only as a critical psychological resource for sustaining long-term careers in high-acuity surgical environments but also as a core correlate of job crafting behaviors. In the operating room context, a strong professional identity is associated with mitigated burnout resulting from prolonged sterile procedures and surgical team pressures, correlates with bolstered confidence in leading complex cases, and is concomitant with fostered willingness to innovate in surgery-specific practices, such as developing new counting protocols, mentoring trainees in specialty teams, and improving surgeon-nurse collaboration (Sun et al., 2024).

Therefore, nursing managers should foster specialty pride among senior operating room nurses by implementing credentialing programs and expanding leadership opportunities. This can include creating tiered technical certification pathways, designating formal Clinical Expert roles, and establishing an OR Innovator program that enables senior nurses to trial new techniques and share outcomes during hospital grand rounds. Publicly recognizing their critical contributions to surgical safety and excellence reinforces their professional value and solidifies their expertise as integral to the operating room team identity.

### 4.3 Limitations

This study has several limitations. First, the convenience sampling method restricted to four hospitals in Zhongshan may introduce selection bias. Consequently, the findings are most representative of the specific context of these participating hospitals, and their generalizability to other geographic regions or diverse surgical environments remains uncertain. Second, the significant overrepresentation of female nurses in our sample inherently limits the cross-gender applicability of the results. The findings primarily reflect the experiences of female operating room senior nurses, and their relevance to male nurses requires further investigation.

Future research should pursue several directions to advance these findings. First, longitudinal studies are needed to track the impact of other influential factors on job crafting among senior nurses. Second, qualitative methods could be employed to further elucidate the complex interactions between intraoperative pressures, professional identity, and adaptive job crafting behaviors. Lastly, future studies should aim for multi-center sampling across diverse regions and strive for balanced gender representation to significantly enhance the external validity of the findings.

## 5 Conclusions

This study provides a theoretical basis for enhancing the job crafting abilities of operating room senior nurses within contexts similar to the participating hospitals in Zhongshan and offers practical guidance for optimizing organizational support strategies and strengthening professional identity in surgical settings. These efforts can promote their career development and contribute to the stable construction of the surgical nursing workforce. However, the conclusions are primarily grounded in data from a female-dominated sample within a specific regional healthcare context. Future studies should expand the sample size, conduct multi-center longitudinal surveys across diverse geographic and healthcare settings, strive for better gender balance, and employ qualitative methods to deeply explore the influencing factors and dynamic evolution of job crafting among operating room senior nurses, thereby enabling multi-dimensional improvements.

In addition, future interventions could include tailored job crafting workshops that guide senior OR nurses in proactively shaping their roles to align with personal strengths and professional goals. Integrating mentorship programs can further enhance role meaningfulness and autonomy. Hospitals may also develop dynamic feedback systems that allow senior nurses to regularly contribute input on workflow improvements and policy changes, reinforcing their agency and organizational value. These evidence-based strategies can collectively foster a supportive and adaptive work environment that sustains both individual wellbeing and team effectiveness in the operating room.

## Data Availability

The data analyzed in this study is subject to the following licenses/restrictions: This dataset is part of an ongoing intervention study and therefore is not suitable for providing raw data. Requests to access these datasets should be directed to Qiwen Lian, 2059202645@qq.com.
